# Book Reviews

**Published:** 1814-09

**Authors:** 


### CRITICAL ANALYSIS
### *OF RECENT PUBLICATIONS*
IN THE
### DIFFERENT BRANCHES OF PHYSIC, SURGERY, AND MEDICAL PHILOSOPHY.

---

*Observations on the Nature and Treatment of Consumption; addressed to Patients and Families.* By CHARLES PEARS, M.D. F.L.S. &c. &c. 8vo. pp. 63. Highley and Son. 1814.

A BOOK may be bad in two ways: it may, by a specious title, induce people to read it, who go on from page to page, hoping the next may turn out better, till they happily arrive at the end, and then find they have lost their time; it may be bad from the errors it contains, and the wrong principles which it promulgates. We conceive the work before us offends in both these respects. All the information in it is taken from writers whose works are much better known than can ever be the fate of this publication, while that portion which the author may claim as being peculiarly his own, is calculated to mislead weak persons, and bias uninformed minds with fatal prejudices in the treatment of a most universal and destructive disease. To medical readers it will only prove injurious from taking up their time; to the public it may occasion the most serious mischief, and on that account we do not shrink from noticing it.

We doubt not the author has seen the disease, he appears to have read upon it, and we will not dispute his opinion respecting the causes which influence it, nor differ with him in considering it as a disease of debility. But here we stop; he has chosen to omit altogether any notion of the disease ever being in its commencement of an inflammatory nature; he seems to have taken his stand nearly at the termination of the complaint; he has confounded the symptoms together in one general mass, which he calls debility, and triumphantly asks, "How then can debilitating means ensure recovery?"—"Is an *increase, continuance*, and *extension* of those means which have induced the disease, a very probable or natural mode of removing it?" He continues in this strain, enumerating symptoms evidently in the last stage of the disorder, and supposing that practitioners really do pursue the most debilitating plan of treatment, bleeding and starving the patient to death. This conduct requires little comment, the author must know, if he has not forgotten, how carefully practitioners attend to the progress of consumption; how frequently they effectually ward off the first attack, by the very means which he so unbecomingly deprecates; how minutely they measure their practice by the symptoms which occur, or which their sagacity anticipates, and that they never either bleed or starve their patient after the suppurative process has commenced; and if they

 ultimately ultimately fail, at least they often have the satisfaction of mitigating the patient's sufferings, and prolonging his life.

The author confidently informs his readers, that "only those recover who have (most wisely and consistently) *disobeyed* the rules by which they were to have been confined." We hardly need state the remedies which Dr. Pears recommends in pulmonary consumption; but in justice to him, and to our preceding observations, we cannot avoid stating the more prominent part of his treatment, which he so strenuously urges. The patient is carefully to avoid milk, and especially asses' milk, it being too weak; and he is by no means to drink weak liquids at any time. But the food is to be of the most nourishing and invigorating kind: "animal food, strong broths, and beef tea, poultry, game, wine, fat, and what is called rear or underdone meat, are the most nourishing and proper; spices, if agreeable, and pickles. Salt and savoury meats may be allowed, and are frequently required by the stomach."—"I (Dr. Pears) frequently find it necessary to allow and order a sandwich of ham, anchovy, red herring, &c. even in the night. The stomach will and does retain this food, when all other is rejected. Also onions, (or a beef-steak and onions,) stewed oysters, &c. with brown stout, or wine."—Wine and water, common draught or bottled porter, good mild ale, home-brewed, and not too new or fermentable, may be drank in general, with the occasional or moderate and regular use of wine, (especially tent-wine,) in small quantity. If they should appear to disagree or excite coughing, (as *every* liquid will sometimes do), repetition and perseverance will soon overcome the difficulty, and prove the advantage thus gained." Between whiles the patient may take an egg beat up with wine, sugar, and milk; in short, the stomach is to be "gradually invigorated by constant but not laborious employ."

Amongst all this cramming there is little room for medicines; and as the author dispatches them in about a page, we suppose he does not place much confidence in any which he has tried; they consist of course in those of the "*cordial, bitter, and tonic kind.*"

Evacuations are reprobated; "of these BLEEDING is the *worst!* When patients are BLED, *their death-warrant is signed with their own* BLOOD."

"When the symptoms arise from *internal* bleeding, as from the nose, lungs, &c. the *same* treatment must be pursued. Avoid *bleeding!* If loss of blood would cure, the very cause of the disease would *prevent* its existence."

Such are the opinions of this author upon the treatment of consumption, which he says are supported by cases already published, the plan being first tried upon himself; "and its success warranted the practice, which has been ever since adopted with an effect beyond every other. He enumerates, amongst his titles, that of "*late lecturer on the structure and management of the human body.*" We presume the want of an audience occasioned the epithet *late* to be prefixed; and, if his discourse upon anatomy was no better than

 this this farrago about consumption, we should conclude he was a disciple of the famous Du Cerf, who offered a celebrated lecturer on anatomy a sum of money for his situation. The professor shewed Du Cerf a bone (an os femoris) which the latter could not name. "How then," said he, "dare you pretend to the first anatomical chair in the kingdom?"—"Oh, sir," replied Du Cerf, "that is nothing; it will not prevent my making a very fine harangue upon it."

---

*An Essay on the Venereal Diseases which have been confounded with Syphilis, and the Symptoms which exclusively arise from that Poison; illustrated by Drawings of the Cutaneous Eruptions of true Syphilis, and the resembling Diseases.* By RICHARD CARMICHAEL, M.R.I.A. President of the Royal College of Surgeons in Ireland, and one of the Surgeons of the Lock Hospital, Dublin. 4to. pp. 121. 1814.

THE importance of the subject of the tract we are about to notice, is too evident to be disputed; and the attention of the profession has been already called to the discrimination of the various diseases which present themselves on the organs of generation, by Mr. Abernethy and Mr. Hunter. The present essay introduces a somewhat novel distinction between syphilis and *venereal* diseases, which, however correct in fact, may lead to some confusion, in as much as they have been considered as synonymous terms. The general plan and purport of the book will be best explained by the author in the following advertisement. The plates in illustration of his cases should have accompanied the work, and perhaps the author and the public might have waited for them, but they will be given in the continuation of the work.

"The object of the following treatise is chiefly to elucidate an important class of diseases hitherto confounded with syphilis, but to which the attention of the profession has of late been attracted by Mr. Abernethy; and it will be satisfactory to state, in the first instance, the opportunities which conduced to enable the author to investigate a subject requiring a very ample field of observation; and, in the second place, to make the reader acquainted with the manner in which that investigation was prosecuted.

"The Lock Hospital of Dublin is probably the most extensive institution in Europe, for the exclusive reception of patients affected with venereal diseases. It is supported by government, and, in general, contains from two hundred and eighty to three hundred patients. The hospital is visited daily by five surgeons, each of whom attends his allotted wards; but the entire institution is, of course, open to the observation of all; so that each has the advantage of witnessing any peculiar or interesting case which may occur in this extensive institution.

"The manner in which the investigation was prosecuted, was on the most most simple plan. Whenever a primary ulcer on the genitals occurred, which was destitute of the characteristics of chancre, *the hardened edge and base*, it was treated without the exhibition of mercury; and the same system was pursued in those cases of constitutional symptoms which had a doubtful appearance. *The scaly syphilitic blotch*, as described in page 129 of Willan on Cutaneous Diseases, and the *excavated ulcer of the tonsil*, as described in page 482 of Hunter,* were alone esteemed to be syphilitic, and treated with mercury.

"As to the affection of the bones: Whenever a patient complained of *nocturnal pains in the shafts of the long bones, or had a decided node or enlargement of the bone*, his disease was esteemed syphilitic, and the use of mercury adopted; but, if the patient merely complained of pains in his joints, or if there was an indication that the coverings of the bone only were affected by an inflammatory swelling, of a doubtful character, an occurrence which was not unfrequent, the employment of mercury was postponed, until the nature of the disease manifested itself by indubitable syphilitic appearances.

"All the cases which did not coincide with these appearances, were carefully noted in the following manner:—

"1st. The appearances on the patient at his admission were marked down; his statement of his complaints, as far as could be collected from him, previous to his admission, was added; and, lastly, the progress and treatment of his disease were noted, in general but once in a week, but oftener, if the symptoms required any change of treatment.

"The cases were noticed before an intelligent class of pupils, and the information contained in the following work, was detailed in general and clinical lectures during the two last winters, in which the nature of the diseases, that have been confounded with syphilis, were elucidated by a frequent reference to the noted cases, and the pupils had opportunities of observing every variety of the symptoms of these diseases on the patients themselves in the hospital, and of contrasting them with those of true syphilis.

"As a number of isolated facts can only acquire importance by leading to general conclusions, so it will be necessary in this work, in order to render it useful, to take a short view of circumstances already known. In the first instance, therefore, a brief view is taken of some morbid poisons, which stand in nearest relation to venereal diseases; under which denomination are included all complaints propagated by sexual intercourse: and the term syphilis is restricted to that disease supposed to be brought to Europe by the followers of Columbus, about the conclusion of the fifteenth century. The symptoms of syphilis are next adverted to; and afterwards, the more immediate object of the work is entered on at large. By

"* The edition edited by Dr. Adams is referred to in this work."

which which preliminary matter, the nature of the pseudo-syphilitic diseases, as they are termed by Mr. Abernethy, and the relation in which they stand to syphilis, and other contagious disorders, will be more clearly understood.

"Some novel, and probably important matter will be found in the chapter which treats of syphilis; and those chapters that relate to the diseases which have hitherto been confounded with syphilis, are altogether the fruits of the author's observations under the plan already explained."

The first chapter is chiefly occupied in detailing the opinions of authors, and the histories of diseases which resemble the syphilitic disease, such as the yaws, sivvens, the Canadian disease of Swediaur, and those anomalous complaints which are said to be produced by the transplantation of teeth; but, as these have often undergone discussion, we shall hasten over them to the more important matter of his work, observing, before we quit this chapter, that in our opinion the author does not, perhaps, quite do justice to the discrimination of the profession in the following remark, for we believe that it is far from being the general practice to treat every sore on the genitals as venereal under whatever shape it may appear.

"Having, as I conceive, adduced sufficient evidence to prove, that ulcers on the generative organs, were at all times common before there was, in this part of the world, any acquaintance with syphilis; and that these ulcers were frequently followed by constitutional disorders; we must acknowledge the necessity of discriminating them from those of true syphilis, and from each other, and not condemn all, however unlike, to a similar mode of treatment, because they happen to be found on the same parts, and are produced by the same kind of communication. We might, with as much consistency, treat all ulcers of the throat alike, whether arising from scrofula, scarlatina, or simple inflammation; yet, strange to tell, at this improved period of surgery, and notwithstanding the valuable observations of Mr. Hunter, Mr. Abernethy, and Dr. Adams, it is very generally the practice to treat every ulcer on the genitals as syphilitic, whatever may be its appearance, character, or distinction."

The characters of the true chancre, as described by Mr. Hunter, and some peculiarities of the appearance as it affects the skin, are well described in the succeeding chapter, as well as the modes of treatment under different circumstances. If the phenomena of chancre are different when affecting the skin from what they are when the glans penis is the seat of the complaint, perhaps the character of the disease arises rather from the peculiarity of the structure affected, than of the affection; and the definition of Mr. Hunter, if this be the case, must fall to the ground, or can only apply to chancre on the glans. This is perhaps worth considering, but we proceed. The treatment which the author recommends in the inflamed phymosis is judicious, and his own. Repeated general general bleeding, and other antiphlogistic means, are used at the same time with mercury. The case which we subjoin may serve to illustrate it.

"Patrick Dunn was admitted into the hospital on the 23d of December, 1811; he was affected with phymosis and profuse discharge; the entire penis was violently inflamed and swelled, a chancre, situated on the external surface of the prepuce, disclosed the nature of the ulcers which were concealed. He complained of severe pain, pulse 120, tongue white and furred, and excessive thirst.

"Twenty ounces of blood were immediately taken from his arm. He was directed to confine himself to bed, to support the penis in the most easy and convenient manner, to make use of emollient fomentations and poultices, and to inject warm water frequently between the prepuce and glans.

"He was also ordered to take a grain of calomel, and to rub in a dram of strong mercurial ointment, night and morning.

"The day following the pain was lessened, but the inflammation and sympathetic fever remained unabated. Sixteen ounces of blood were taken from his arm, and he was desired to persevere in the other means recommended.

"On the third day, the pain, inflammation, and swelling, were considerably diminished. On the fifth day, his mouth was affected, and the chancre on the external prepuce had assumed a healthy appearance. The swelling of the penis was rapidly reducing. He was directed to discontinue the pills, but to persevere in the ointment.

"A strong mercurial action was preserved in the system till the 24th of February, and he was shortly afterwards discharged well."

Some very useful remarks on the distinctions and treatment of bubo-warts, (in which he recommends the acetic acid,) and the venereal eruption, in short, the history of *syphilis*, occupies the rest of the chapter, which we shall pass over, after remarking that the whole of this part of the work displays considerable and judicious observation.

The principal end and subject of the work is pursued in the ensuing chapter; and the author divides the primary diseases, which he calls venereal, and distinguishes from syphilis, into two classes, comprehending six species, as follows:

"In the first class I would include, 1. A superficial ulcer without induration, but with elevated edges. 2. A similar ulcer, destitute not only of induration, but of elevated edges. 3. An excoriation of the glans penis, and internal surface of the prepuce, attended with purulent discharge. 4. Gonorrhœa virulenta. I have not been able to trace any constitutional symptoms arising from the first species. The constitutional symptoms of the other three species are precisely alike, and cannot, in the slightest degree, be distinguished from each other.

"In the second class may be comprised the two remaining species of pseudo-syphilitic disorders, viz. 1. The phagedenic ulcer; and, 2. The

2. The sloughing ulcer. There is a strong resemblance between these two primary diseases, as the sloughing ulcers, when the sloughs separate, can scarcely be distinguished from the phagedenic. Whether their constitutional symptoms are alike, is more than I am willing to decide, not having witnessed more than one case of the constitutional symptoms of the sloughing ulcer; but I should not omit to mention, that the appearances were favourable to the presumption of their similarity. But I have had an opportunity of observing numerous instances of the constitutional symptoms of the phagedenic ulcer, and they are materially distinct from those which arise from the primary affections comprised in the first class."

For a detail of the cases from which the author draws his conclusions and describes his treatment, we must refer the reader to the work itself. We should have been glad that the cases had been pursued to a greater length of time, and more particularly as the author does not hesitate to use *mercurial* topics in the cure of them; and perhaps his conclusion, that, had the cases been followed by any secondary symptoms, the patients would have returned to the hospital, may not be altogether assented to; if even the contrary inference be not drawn, from the irregular habits and prejudices of that class of patients, the inquiry is very important however, and conducted by the author in a very candid and able manner.

The remaining part of the book is occupied on the constitutional symptoms which present themselves in diseases which resemble syphilis, which, as we have said quite enough to excite the attention of the reader to the perusal of a very useful book, we shall defer till we have an opportunity of consulting the plates, in the next part, without which this subject is incomplete, and cannot be fairly discussed.

---

***Experiments on the Production of Animal Heat by Respiration. An Inaugural Dissertation, read and defended at the Public Examination, before the Rev. President and the Medical Professors of Harvard University.*** New England Journal. By Enoch Hale, Jun. M.D.

This is an interesting dissertation, and may be considered as adding some important facts to our present stock of physiological knowledge. The production of animal heat has been a subject of much interest and speculation. Like all other natural phenomena, it is susceptible of more illustration from simple facts and appropriate experiments, than it can receive from the most ingenious and complicated theories. It will be recollected that the experiments of Mr. Brodie, formerly detailed in this Journal, favour the conclusion that no heat is produced by respiration in the lungs. Dr. Hale, in the course of his inquiries, was surprised to meet with results totally different in this respect from those of Mr. Brodie. Instead of finding that an animal in which artificial respiration was kept up, cooled faster than one which did not respire, and the lungs faster than the rest of the body; he found that the respiring animal retained its heat the longest, and that the lungs were the hottest part of the animal. We shall insert at length a part of the author's experiments, with his remarks and inferences on the whole.

*Experiment* 1.—For my first experiment I chose two young dogs of the same age and size. A thermometer in the room at the beginning stood at 66° of Fahrenheit.

I divided the spinal marrow of the dogs between the occiput and atlas, leaving a sufficient interval of time between killing the two, to enable me to observe and note down every appearance in each, that might occur. Immediately after this was done, a small opening was made in the abdomen of each animal, and the bulb of a thermometer inserted, and retained till the mercury became perfectly stationary; when it was withdrawn, and the opening covered with adhesive plaister till another observation was made.

The first animal lay perfectly still without any struggle, from the moment of the division of the spinal marrow. The heat at successive times was as follows:

At the commencement the mercury stood at 96°.

Fifteen minutes after it rose only to 93½°.

In half an hour it was at 92½°.

In forty-five minutes at 91°.

In an hour and five minutes at 89°.

And in an hour and twenty minutes at 88°.

The thorax of this animal was not opened.

Immediately after pithing the second animal, I commenced artificial respiration by means of a common bellows, provided with a double tube, (which was inserted into the trachea,) so contrived as to expire the air that had been breathed, without its passing into the bellows. In the course of the experiment, some of the expired air was passed through lime-water, which it rendered turbid.

As soon as the respiration was begun, the animal had pretty violent contractions of the voluntary muscles, which frequently returned till near the end of the experiment.

At the commencement, the thermometer in the abdomen stood at 96°. The heart was felt through the ribs beating from one hundred and thirty to one hundred and forty times in a minute.

Fifteen minutes after, the pulsations of the heart continued vigorous, and as frequent as at first. The thermometer stood at 94°.

In half an hour, the pulsations continued the same. The thermometer was at 93°.

In forty-five minutes, no change in the pulsations. The thermometer was at 92°.

In an hour and five minutes, the pulsations were about as frequent, and nearly as strong, as at first. The thermometer stood at 91°.

In an hour and twenty minutes, the pulsations had grown so feeble as to be but indistinctly felt through the ribs. The heat in the abdomen was 90½°.

I now

I now opened the thorax, and immediately placed the thermometer in contact with the lungs, where it stood at 91½°. I then laid open the pericardium, and put the bulb of the thermometer in contact with the heart, where it fell to 90½°. This surprised me, and lest it might possibly be occasioned by the presence of external air after the thorax was opened, I carried the thermometer a second time to the lungs, when it evidently rose a full degree.

Blood oozed out from the small vessels as I cut into the fleshy part, nearly as much as in a living animal.

The left side of the heart and the pulmonary veins were filled with florid blood, and the right side and venæ cavæ with dark-coloured blood. The heart continued to contract for some time after the thorax was opened.

Finding it extremely difficult to carry on a perfect respiration without a better apparatus, I procured a double bellows, so constructed that while one part filled with fresh air from the atmosphere, the other filled by exhausting the lungs of that which had just been thrown into them. As the bellows closed, the fresh air was thrown into the lungs, and the respired air into the atmosphere. With this bellows the subsequent experiments were performed.

*Experiment* 4.—The temperature of the room was 71°. Two small animals of the same age and size, whose pulsations were one hundred and twenty in a minute, were killed by dividing the spinal marrow; and their temperature observed every fifteen minutes, by inserting a thermometer into an opening in the abdomen. Effectual care was taken that the thermometer should be affected by nothing but the temperature of the animal. Between the observations, the opening was kept closed with adhesive plaister.

The pipe of the bellows was inserted into the trachea of the first animal, and respiration commenced immediately after it was pithed. At this time the thermometer in the abdomen stood at 98°. The inspirations were repeated forty times in a minute, and with such force as to imitate natural respiration as much as possible.

Soon after I dissected into the neck, and divided the nerves going from the head into the thorax, without injuring the large blood-vessels.

There were several violent contractions of the voluntary muscles, during the whole course of the experiment; but they were less frequent in this than in some preceding ones.

Fifteen minutes after pithing the animal, the thermometer stood at 96½°. The heart beat as strong as at first, one hundred and twenty times in a minute.

In half an hour the thermometer was at 94¼°. The pulsations of the heart were still the same.

In forty-five minutes the thermometer was 92°. The pulsations were one hundred in a minute, and rather more feeble than at first.

In an hour the thermometer was at 90°. The action of the heart was diminished to eighty-four pulsations in a minute, not quite so strong as before, though still very distinctly felt through the ribs.

I now began to open the thorax; but the pipe of the bellows, just at this time, slipping out of the trachea, engaged my attention, so that I did not get the thermometer fairly to the lungs till eight minutes after. It then stood at 89°. The temperature of the heart was about the same.

Blood flowed from the small vessels, as I cut into the fleshy parts. The heart continued to contract for some time after the thorax was opened, and irregularly after the respiration was stopped.

The other animal gave no visible signs of life, except for the first moments after it was pithed. The thermometer in the abdomen at first stood at 98°. In fifteen minutes it fell to 96°. In half an hour to 92½°. In forty-five minutes to 88°. And in an hour to 85½°.

An hour and eight minutes after the animal was killed, the heat of the lungs was 88½°.

*Experiment* 5.—The temperature of the room, the first part of the time, was steadily 68°. Two small animals, of the same age and size, were killed, as before, by dividing the spinal marrow; and the process of cooling observed every fifteen minutes, by an opening in the abdomen.

In the first animal, respiration was commenced as soon as possible after it was killed. The respiration was compared with that of the living animal, and made to imitate it pretty exactly.

No blood was lost in pithing the animal. It had entirely ceased struggling before the pipe of the bellows was fixed in the trachea; but upon the first inspiration the muscles acted violently. While this was doing, an assistant placed the thermometer in the abdomen, and found the heat 102½.*

The circulation went on as perfectly as could be wished. Not the slightest failure of the action of the heart could be perceived, either in force or frequency, for the first hour and a half.

During the first half or three quarters of an hour, the contractions of the abdominal muscles were so violent, as frequently to force out the intestines at the opening made for the thermometer, notwithstanding the attempts to keep it closed with adhesive plaister. Finding these attempts unavailing, I at length entirely closed the opening with ligatures, and made another very small one, which was also closed in the same way, except during the observations. This made the animal cool much faster at first than afterwards.

In fifteen minutes, the thermometer stood at 100°.

In half an hour, it was at 97°.

In forty-five minutes, at 95½°.

In an hour, at 95°.

Five minutes after this, there was a copious evacuation of urine. There had been none, as is common, when the animal was killed, though there was an evacuation of fæces.

---

* The thermometer used in this experiment stood one degree higher than that used in the preceding. The difference was constant, in all variations of temperature.

An

An hour and fifteen minutes from the beginning, the thermometer stood at 94½°.

In an hour and a half, it was 94°.

In an hour and forty-five minutes, it was at 93¼°. The pulsation of the heart was now, for the first time, observed to be a little more feeble.

In two hours, the pulsation was much as at the last observation. The thermometer stood at 92°. I now opened the thorax, and applied the thermometer to the lungs and to the heart. The temperature of both was 92°.

The blood flowed freely from the small vessels, as I cut them. The arterial system was filled with florid, and the venous with black blood. The heart continued its action some time after the respiration was stopped.

The other animal was not killed till the afternoon of the same day. The thermometer in the room had then risen to 71°, and towards the conclusion to 73°.

The spinal marrow was divided, and the animal suffered to lie, as in the preceding experiments.* It exhibited no signs of life. The thermometer in the abdomen stood at 102½°.

In fifteen minutes, it was at 100½°.

In half an hour, at 98¾°.

Thirty-five minutes after killing the animal, I introduced the pipe of the bellows into the trachea, and began respiration. This was continued to the end of the experiment, with the same frequency and force as had been used in the respiration of the other animal. No visible symptom of life was revived by it.

In forty-five minutes, the thermometer was at 96°.

In an hour, at 94½°.

In an hour and fifteen minutes, at 92°.

In an hour and a half, at 90°.

In an hour and forty-five minutes, at 89°.

And in two hours, at 86°.

I now opened the thorax. The heat of the lungs was 81°. The lungs were very full of blood, which, of course, was florid. Both sides of the heart were filled with black blood. I could discern no appearance of there having been any circulation.

From the foregoing experiment, I think it clearly appears, that sensible heat is, either directly or indirectly, produced by the respiration of animals, after the communication is cut off between the brain and the rest of the body. This heat is nearly in proportion to the effectiveness of the respiration in carrying on the circulation, and in producing the changes proper to the living state.

In the first experiment, notwithstanding the constant influx into the lungs of air more than 20° colder than the animal, the respiring animal, at the end of an hour and twenty minutes, was two degrees

---

* It was my intention to have carried on respiration in this animal with carbonic acid gas; but my local situation rendered it impossible to acquire even the simple means necessary to obtain the gas.

and a half warmer than the other; and the lungs were one degree warmer than any other part of the body. This last circumstance was probably occasioned by the effects of respiration being still produced in the lungs, after the vital powers were too much reduced to eliminate the heat in the different parts of the body.

The second experiment, taken by itself, proves but little; the respiring animal cooling faster than the other. But when viewed in connexion with the third, its importance is very considerable. For although the respiration was so imperfect that the action of the heart, produced by it, never exceeded seventy-six pulsations in a minute, and much of the time was hardly perceptible through the ribs, yet it was sufficient to keep the temperature of the lungs equal to that of the rest of the body, for the space of an hour and twenty minutes, when breathing an atmosphere almost fifty degrees colder than the animal; whereas, in the third experiment, with the lungs inflated in the same manner, in the same temperature of the atmosphere, the lungs cooled nine degrees more than the abdomen, in only twenty-five minutes, although here there was also an action for a short time capable of producing heat, as appears by the slow cooling of the animal at first.

The fourth experiment gives a result still more decisive. The respiration being more perfect, we find, at the end of an hour, the respiring animal four and a half degrees warmer than the other. In this and the second experiment, the lungs of the non-respiring animals were considerably warmer than the rest of the body. This is precisely what we should expect, when we consider that air *confined*, (which is the state of the air in the cells of the lungs,) is one of the slowest conductors of caloric known.

The fifth experiment shows the effect of inflating the lungs, without the changes answerable to those of the living state. I have only to regret the accidental protrusion of the abdominal viscera, which cooled the respiring animal so much at first. But, notwithstanding this accident, the temperature of the lungs and abdomen of this animal, at the end of two hours, had fallen only to 92°; while in a warmer atmosphere, the abdomen of the other animal, in the same time, fell 6°, and the lungs 11°, lower, being only 8° above the surrounding air.

It is a matter of regret to me, that Mr. Brodie's results are totally different from those I have obtained. One cause may perhaps be, the loss of substance occasioned by separating the head of the respiring animals. As this was not necessary to a complete destruction of the nervous connexion of the brain with the body, I did not do it. In one or two instances, I divided the nerves of the neck; in others, I only divided the spinal marrow.

The separation of the head, however, is not sufficient to account for all the difference between Mr. Brodie's experiments and mine; and, I confess, I know not how to reconcile them. I will only observe, therefore, that my experiments were begun under a strong persuasion that the contrary from what now appears was true. They have all been performed in the presence of respectable gentlemen, tlemen, who were uninfluenced by any opinion I might afterwards adopt, and who were witnesses to the faithful record of every leading fact that occurred.

The correctness of Dr. Hale's experiments and inferences will derive no small support from the following recent statement.

Mr. Brodie, Member of the Royal Society of London, has attempted to ascertain the state of the temperature and secretions in animals that are kept alive (by inflating the lungs) after being decapitated. I have, says M. Le Gallois, repeated the experiments of this author so far as relates to the temperature. It has not appeared to me that the results which he announces are so uniform as he states. Mr. Brodie assures us, that decapitated animals, which are kept alive, cool as fast as if they were dead. It is true that they cool considerably; but I have always found that young cats cool less than after death. The difference in my experiments has been from 1 to 3 centigrade degrees. In rabbits it is generally somewhat less.

---

*Observations on those Diseases of Females which are attended by Discharges*; by CHARLES MANSFIELD CLARKE, Member of the Royal College of Surgeons, &c. Longman and Co. 8vo. pp. 304.

(Continued from p. 162.)

THE vaginal discharge Leucorrhœa, more familiarly known by the terms whites, or weakness, is considered in the second chapter; the latter term is properly reprobated, as leading to an improper mode of treatment.

"It is very important to inquire into the cause of these discharges; by the knowledge of which, the judgment of the practitioner will be directed to the best mode of treatment.

"If the discharge is the effect of weakness, and if by its continuance the original weakness is increased, tonics will be required. If it depends upon some tumour in the vagina, the removal or support of this will also remove the discharge. If it arises from inflammatory action, this must be removed before any endeavour to restrain it is employed; for, as the discharge during its continuance lessens the violence of the disease which produced it, it should not be checked till such inflammatory action is diminished. Nothing can be more injurious under such circumstances, than the exhibition of tonics and stimulants, as cantharides, turpentine, and steel.

"In many cases it is as injurious to restrain the discharge from the parts, as it would be to put an end to the natural salivation of a teething child whilst the determination of blood to the head continues, or to heal an ulcerating surface in a constitution which has been long accustomed to it, without substituting some other secretion for it."

"There are mixed cases, where the discharges vary from their usual appearance; moreover, a discharge of one kind will mark one stage of a disease, and a discharge of another kind a different stage.

As

As happens in diseases in other parts of the body, one disease also is sometimes blended with another, and the discrimination of these modifications and irregularities constitutes no small part of the skill of the practitioner. A schirrhous tumour of the uterus may have been attended (for years perhaps) by an increased secretion of simple mucus; but upon this disease becoming active, by inflammation attacking the tumour, so as to convert it into cancer, the discharge becomes purulent and highly irritating. The period of this conversion is indicated by the alteration in the nature of the secretion.

"In the cauliflower excrescence of the os uteri,* the discharge consists of little more than a clear watery fluid: blood, however, is sometimes mixed with it, or perhaps comes away alone in large quantities. Nevertheless, the discharge of blood forms no part of the peculiar character of this disease, but it is generally produced by violence or improper exertion.

"The discharges from the vagina may be comprised under the following heads:—1. Transparent mucous discharge.—2. White mucous discharge.—3. Watery discharge.—4. Purulent discharge.—5. Sanguineous discharge."

"Of some diseases of the uterine system, the white mucous, and the watery discharge, are pathognomonic symptoms; but the sanguineous belongs to none exclusively, being met with occasionally in most of them."

In the fourth chapter the doctrine of Sympathies is explained. Uteri affectus omnes fere ventriculo nocent.—Heberden Comment. de Historia Morbor. cap. 97.

"In cancer of the uterus, the stomach is always more or less affected with vomiting. When the uterus has been ruptured, vomiting comes on; and the matter rejected is of a black colour, resembling coffee-grounds."

A case is related which illustrates this subject. "A lady between fifty and sixty years of age, was attacked with pain in the back and at the bottom of the belly, attended by a purulent discharge from the vagina: there was nausea and vomiting, spasmodic pains were referred to the epigastric region, and there was pain over the anterior part of the head. An examination being made, the uterus was found extremely sensible to the touch, but it was not enlarged; at least no enlargement could be ascertained by examination: recourse was had to the hip bath and other remedies, and at the end of a few days the pain in the back and belly ceased, the sickness went off, and the patient was no longer troubled by head-ache. At various times since the first attack, this patient has been liable to the same symptoms, which have come on in the same order of succession; and they have yielded to the same means.

---

* "See a paper by Dr. Clarke, in vol. iii. of the Transactions of a Society for the Promotion of Medical and Chirurgical Knowledge.

"Pain

"Pain in the lower extremities attends some uterine affections: previously to the appearance of the menses, and before the coming on of each period of menstruation, it is experienced by many women. It has been observed as a precursor of puerperal mania.—This pain in the legs is very different from cramp in the lower extremities, produced by pressure upon the sciatic nerve of one or both sides; and it takes place in cases where no such pressure is, or can be made.

"The diaphragm is apt to be affected in some diseases of the uterus, so that the patient becomes subject to hiccough.

"The mind also sympathizes with the uterus. This it does always when the stomach is affected by disease: but this is to be considered as one of the compound sympathies; for, both in men and women, when the digestive organs are disordered, the faculties of the mind are apt to be enervated; and occasionally to so great a degree, as to incapacitate the patient for attending to common business, or for enjoying the ordinary pleasures of life.

"But besides this affection of the mind, induced through the medium of the stomach, many cases are found where the connexion subsisting between the uterus and the brain appears to be more direct; as in furor uterinus, puerperal convulsions, and in those cases of madness which succeed parturition, when there is little of bodily disorder. The connexion between the uterus and the sensorium may account for the greater number of instances of madness which occur in females than in males; it appearing that the number of women, compared with that of men, affected by madness in this country, is in the proportion of five of the former to four of the latter.*

Among the diseases attended by mucous discharges from the vagina, are some which consist in the displacement of parts. Procidenti uteri, procidenti vesicæ, and inversion of the womb.

Chapters five, six, and seven, are devoted to the consideration of procidentia uteri, and its treatment: a disease, in its advanced state, productive of great distress to the patient, and often permanently incurable. The more important part of these chapters is the description of the symptoms characteristic of this complaint, which is particularly incumbent on the practitioner to make himself acquainted with in its *early stages*, as in its commencement, in a great majority of cases, it is susceptible of cure.

The author observes, "at the commencement of this ailment the woman complains of pain in the back, and this symptom sometimes continues for a great length of time without any other: pain is also felt in the groins, extending towards and terminating in the labia: there is a sense of fulness in the parts, and an increased discharge of transparent mucus from the vagina. As the disease proceeds, the pain in the back is described as the pain of dragging: the patient now has a sense of bearing down, or of weight; feeling, as she expresses it, as if every thing was dropping through her. The dis-

* See Haslam's Observations on Madness and Melancholy.

charge increases in quantity. The pain in the groins arises probably from the round ligaments being stretched, and that in the back perhaps from an elongation of the parts connecting the uterus behind. As soon as the erect posture is changed for the recumbent position, these symptoms go off.

"Strangury, although not a constant attendant, sometimes is present, and annoys the patient until the procidentia is cured. A lady, whose constitution was weak, and who had borne several children, was attacked by pain in the groins; she had a discharge of mucus from the vagina, and was affected by a frequent desire to make water, voiding very little at each attempt. She had employed poppy fomentations and opium, and had taken some oily purgatives, without experiencing the least good effect. Upon further inquiry it appeared, that the pain in the groins left the patient at bed-time, and that at the same time the frequent inclination to make water went off. This led to an examination of the parts, by which a procidentia uteri was discovered. The whole plan of treatment was now changed. She used an astringent injection, took some cinchona with sulphuric acid, and confined herself to the sofa. By pursuing these means, the strangury and all the other symptoms left her as her strength was restored, without the use of any mechanical means.

"The pain in the back which attends procidentia of the uterus, should be distinguished from that which is met with in cases of separation of the joint between the os ilium and the os sacrum, after some cases of labour. It has been remarked, that the pain in the back arising from procidentia is greatest when the patient is erect, and that it subsides in the horizontal posture. In the case of separation of the joints alluded to, the patient has a great difficulty in standing, or perhaps cannot stand at all, is uneasy even in the recumbent posture, and incapable of moving in bed without great pain."

These two complaints may exist together, as the author illustrates by a useful case. The separation of the bones, in this instance, was strikingly relieved by a leathern belt round the body.

"The symptoms arising from the sympathy between the stomach and the uterus are very distressing. The appetite becomes irregular, or is totally lost: the stomach and bowels lose their tone, and there is a great sense of distention in the belly arising from air, which may be heard when moving from one part to another; the spirits flag; every employment becomes irksome, and life itself is considered as scarcely desirable. The diaphragm is sometimes affected by spasm, and hiccough is produced."

When the vagina is long exposed to the action of the air, it is observed to go into ulceration. "This does not attack the whole of the exposed surface at once; but small spots or patches inflame and ulcerate, and these sometimes run into each other, but the whole surface is seldom covered by them. These ulcerations are generally not deep, and they have the appearance of healthy sores."

It should not be forgotten, that, when the uterus has descended so low as to be external to the vagina, its characteristic mark is the os uteri at the lower part of the tumour; and that, in all examinations, tions, they should be made while the patient is erect. It will be obvious also, that the symptoms are much diminished when the woman is in a recumbent posture.

The mode of treatment recommended is that which is generally employed; support to the womb by pessaries, cold astringent injections, tonics, &c. which are varied according to the several indications. The best pessaries are those of box wood. Occasionally the oval one is found preferable " in those cases in which the tone of the vagina is so very much diminished as to make a large support necessary; because in this case the oval pessary rests by its two extremities upon the sides of the vagina; but lying with its long diameter applied to the short diameter of the lower aperture of the female pelvis, it neither interferes with the rectum nor with the urinary passage."

Where laceration of the perineum is present, the ordinary pessaries cannot be retained; it becomes then necessary to have recourse to the globular, which, if passed up the sacrosciatic ligaments, will sufficiently contract the lower aperture of the pelvis to enable it to be retained.

Procedentia vesicæ, or according to some procedentia vaginæ, is very rarely relieved; the former name is preferred, because it directs the mind of practitioners to an important part of the treatment. It will be obvious, that in this case the part is more uncomfortable in proportion to the quantity of urine in the bladder. A peculiar symptom which marks this complaint, is a pain referred to the navel, with sensation of tightness, which is also proportioned to distension of the bladder: this may probably be accounted for by the known attachment of the superior ligaments of the bladder to the navel. It is obvious this pain will not be relieved by internal remedies. In procidentia vesicæ the stomach is less frequently affected than in procidentia uteri; in the latter there is an opening in the lower part of the tumour, which is not the case in procidentia vesicæ. Encysted tumours may sometimes be mistaken for prolapsed bladder; but they are never diminished in size, like the latter, by the expulsion of its contents.

" Procidentia vesicæ admits of remedy by the use of a support introduced into the vagina; and the hollow pessary of a globular form before mentioned, is more serviceable than any other.

" Hollow pessaries made of the shape of an egg are worn by some women more comfortably than the globular pessaries; particularly in those cases where the diameter of the vagina is but little increased by relaxation; where the length of the pessary, from the perinæum to the falling portion of the bladder, must of course be sufficient to support the latter; and where, if the instrument were globular, unnecessary pressure and pain would be the consequence.

" The globular and the oviform instrument should be provided with four holes (in the latter, at the broad extremity), through which two pieces of silk can be passed, by means of which the instrument may be occasionally withdrawn by the woman or practitioner. Two holes would be sufficient for this purpose, if the

 strength strength of the silk could be depended upon; but as it may happen to break by the force employed in withdrawing the instrument, the remaining sound piece affords the means of bringing it away. For want of this precaution, women who have worn the oval pessary have had some difficulty in removing it when the tape has been broken.

"Solutions of astringent substances should be thrown into the vagina often in the day. Particular care should be taken to avoid straining, as every exertion of this kind must affect the displaced part, and may force away the instrument. The woman should therefore avoid lifting heavy weights; and the bowels should be kept in such a relaxed state, that the fæces can be passed without any great exertion: the woman may therefore eat freely of fruit and vegetables; and, if any assistance is required from medicine, the mildest purgatives are to be preferred. As the urinary bladder, at different periods, contains very different quantities of urine, and as the degree of the procidentia will depend upon the degree of distention of the bladder, especial regard should be had to prevent the accumulation of urine, by desiring the woman to make water frequently."

Procidentia vaginæ is not attended with the constitutional symptoms of the procidentia vesicæ, nor the local inconveniences of procidentia uteri. The term implies a relaxation of the posterior part of the vagina.

The complaint may, among other obvious causes, be produced "by cysts belonging to diseased ovaries falling down into the hollow between the rectum and the posterior part of the vagina. In one case where this happened in labour, the author was consulted, under a supposition that the prolapsed part was the bag of membranes formed by the amnion and chorion, and attempts had been made to break them. The case was terminated by opening the child's head, by means of which operation the life of the woman was saved. After the labour the cyst went up again into the cavity of the abdomen, and the vagina being no longer pressed down, regained its natural situation.

"No effect in this disease is produced upon the shape of the os uteri, because the cervix of the uterus is hardly at all connected to the rectum, and the cellular membrane between the vagina and rectum is very loose, and readily admits of the vagina projecting."

The treatment consists in keeping the rectum empty, by mild aperients, and in supporting the prolapsed by a globe pessary. The use of astringent injections will also be found serviceable.

"*Inversio uteri.*—This complaint consists, as the name imports, in an inversion of the cavity of the uterus, so that the fundus comes through the os uteri.

"In the present improved state of the art of midwifery, this disease is very seldom met with, because it is generally a consequence of mismanagement of the placenta.

"Women who do not bear children are, for the most part, exempt from this complaint; but it is said that it may be produced by by the weight of a polypus attached to the fundus of the uterus. This cause may of course render unmarried women the subjects of the disease; but it will be rarely met with: first, because polypus itself is unfrequent; secondly, because the polypus must be very large and heavy, that it may have the power of drawing down the uterus; thirdly, because an unimpregnated uterus is unyielding and firm; and fourthly, because the polypus, to produce the effect, must be attached exactly to the fundus of the uterus. In labours which have been badly conducted, the uterus is in a much more relaxed state that when the management has been judicious.

"The immediate consequences of an inverted uterus, when it takes place after delivery, are hemorrhage, faintness, and a sense of fulness in the vagina. The woman in this case compares the feeling with the sensations which she experienced just before the child was born. If the nature of the accident is discovered early, it will admit of a ready cure, by the return of the parts to their original state. This is to be effected by making pressure upon the LOWER PART only of the tumour, so as to cause this part to be received into that above it: a continuance of the same pressing force will in some cases quickly reduce the tumour. If the uterus has not been long displaced, and is much relaxed by loss of blood, the operation will be proportionably less difficult. The author has been called by other practitioners to cases of this kind, where the patient has expired, in consequence of hemorrhage, before the nature of the accident has been ascertained, when the uterus is inverted, and the placenta still adhere."

In the chapter on hemorrhoids, the author remarks, that ulceration frequently takes place on their surface, and that the sores often resemble in appearance those which arise from venereal infection. Similar sores are also observed upon the sides of the anus, to the distance of two inches or more; and they are likewise formed within the labia. Those on the outside of the anus resemble venereal sores; for the cellular membrane is absorbed more quickly than the skin, which gives an appearance of high edges to them. They also become very difficult to heal: but these ulcerations differ from venereal sores, in not having their surfaces covered with that thick yellow film which is met with in chancres; and although the sores do sometimes run into each other, yet they do not spread with the same rapidity as chancres. Besides, chancres will heal by the use of mercury: this will rather be injurious in the other ulcer, which will heal by the application of simple stimulants, such as solutions of sulphate of copper, or of nitrate of silver.

"A discharge of mucus from the vagina is a concomitant symptom of the piles; for the internal iliac artery supplies both the hemorrhoidal vessels and those about the vagina with blood; and it will be found difficult to restrain this discharge whilst the hemorrhoidal tumours continue.

"When a tumour in the cavity of the pelvis occasions the disease, the symptoms will continue till the tumour rises into the cavity of the abdomen. If that tumour should be a pregnant uterus, quickening ing will cure or relieve the patient, when the piles occur in the early part of pregnancy; and labour, when they appear in the more advanced stages. But, if the pressure should be caused by any morbid tumour, as the progress of it is very uncertain, as it sometimes proceeds very rapidly and sometimes very slowly, it will be difficult to foretel how long the pressure will continue."

Ascarides are said to be another cause of mucous discharge from the vagina, attended with extreme and insufferable itching.

" *Carcinoma Recti.*—The whole circumference of the gut is most commonly affected; and, the parts becoming thickened in consequence of the disease, the capacity of the canal is diminished, and the passage of the fæces through it is impeded. The resistance produced by this cause makes the discharge of the fæces very painful; and as piles are a very common disease, and are generally supposed by patients to be the cause of any pain or difficulty in voiding the fæces, this complaint has been mistaken for them, and the patient has suffered the inconvenience without being aware of any danger.*

" In consequence of this narrowness and obstruction in the rectum, the colon becomes gradually more and more distended; and, upon an inspection of the body after death, it has been found to contain several pints of fluid, resembling a mixture of fæces and water.

" This disease has also been mistaken for stricture of the rectum; and the patient has been much injured by the mode of treatment pursued in that disease, and her death has been accelerated. Bougies introduced into the constricted part have produced a great degree of inflammation in the neighbourhood; and thus, by adding to the thickening of the gut, increased the symptoms which they were intended to alleviate.

" The pain attending the complaint is of the darting or lancinating kind; and, being referred to the neighbourhood of the uterus, has led to a supposition that the uterus was the diseased part. This error, with regard to the seat of the complaint, if its true nature is understood, is not very important; because, whether the complaint is in one viscus or the other, the principles upon which it is to be treated are the same.

" In carcinoma of the rectum the pain will be greatly increased by the passage of the fæces, and the pain will be such as the patient would feel upon the rough handling of any external tumour of a similar character; whereas in the common stricture of the rectum, although there may be pain, it will be by no means so acute, being

---

* In speaking of the treatment of piles, the author seems to have forgotten a most invaluable remedy, commonly known by the name of Ward's Paste. We are in the habit of preparing this medicine, in the form of powder, thus;

℞. Enulæ Camp. ℥ij.

Sem. Fœnicul, ℥ iij.

Piperis Nigri, ℥j.

A tea-spoonful should be taken three times a-day in honey.

occasioned occasioned merely by the resistance offered to the passage of the contents of the gut.

"In carcinoma of the rectum, acute pain is occasionally felt when no endeavour is made to expel. In stricture of the rectum, the pain is felt only at this time, or for a short time afterwards.

"The constitution also is more likely to be affected in carcinoma than in stricture of the rectum; and the sympathies between the part diseased and other parts will be more likely to be excited in carcinoma than in stricture.

"The hemorrhoidal veins are apt to become enlarged, and sometimes to bleed. The bleeding may have some effect in retarding the progress of the complaint. Small œdematous tumours about the anus are also very liable to be formed; but both of these symptoms are likely to be met with in other diseases.

"When the uses of the rectum are considered, and its liability to be stimulated, it will appear probable that carcinoma of the rectum will advance with greater rapidity to the more active stages of the disease, than when it attacks parts less exposed to pressure or disturbance; but upon this subject it will be difficult to form any precise opinion, because it is impossible to know how long the disease may have existed before the practitioner was consulted; and it frequently happens that he is not consulted at all until the inflammatory action has commenced, which attends the conversion of the complaint into the ulcerated state. Moreover, the disease not being frequent, opportunities of collecting information respecting it will not often occur.

"That the mesenteric glands are affected in the latter stages, may be learned from writers on morbid anatomy. Dr. Baillie states, that when a portion of the intestinal canal becomes cancerous, some of the absorbent glands in the mesentery also become affected with the same disease, in consequence of the matter of cancer being conveyed to them by the absorbent vessels. This explains the great emaciation which commonly attends the disease. The mere irritation and pain, and the quantity of the mucous discharge from the vagina, during the first stage of this disease, may in some measure account for it; but if the parts concerned in the conveyance of the chyle into the blood have their structure likewise altered, it is reasonable to expect that the emaciation and loss of strength will be more quickly produced.

"When the disease becomes cancerous, the symptoms begin to be more formidable, the mucous discharge is converted into one of a purulent kind; but the history and treatment of this stage will be considered under the head of Purulent Discharges. It may here, however, be remarked, that occasionally in the ulcerated stage a communication is made between the rectum and the vagina, in consequence of the destruction of the parts which naturally separate these cavities from each other; that the pain becomes more acute; that the stomach is apt to be affected with vomiting; and that hectic fever sometimes supervenes."

